# Use of Ecological Momentary Assessments to Evaluate Alcohol Use With American Indian Women

**DOI:** 10.1111/acer.70445

**Published:** 2026-11-06

**Authors:** Jessica D. Hanson, Rebecca J. Gilbertson, Cayden Kleffman, Amy Versnik Nowak, Megan Charboneau, Michael D. Evans, Scott Engel, Marcia O'Leary

**Affiliations:** ^1^ University of Minnesota Duluth Duluth Minnesota USA; ^2^ Missouri Breaks Industries Research Inc. Eagle Butte South Dakota USA; ^3^ Clinical and Translational Science Institute University of Minnesota Minneapolis Minnesota USA; ^4^ Sanford Biobehavioral Research Fargo North Dakota USA

**Keywords:** alcohol, American Indian women, ecological momentary assessment

## Abstract

**Background:**

Understanding the factors surrounding alcohol use among American Indian women requires real‐time, event‐level data collection. Ecological momentary assessment (EMA) offers a promising approach to better understand the context around drinking in this population. The goal of this study was to utilize EMA to track daily alcohol consumption, the setting in which alcohol consumption occurred, and affect among American Indian women over a 30‐day period.

**Methods:**

Following formative think‐aloud sessions to refine survey instruments, 74 American Indian women (ages 18–50) who reported recent alcohol use completed baseline assessments, including the Timeline Followback (TLFB), the Alcohol Use Disorders Identification Test (AUDIT), and measures for depression (CES‐D‐R‐10) and social support (ISEL). Participants were then enrolled in a 30‐day EMA protocol receiving three daily text prompts via the ReTAINE system to report on alcohol quantity, beverage type, social contexts, and mood.

**Results:**

Of the 74 American Indian women who completed baseline data, *n* = 40 participants completed at least 35% of the EMA prompts, yielding 2278 records across 359 drinking days. Binge drinking throughout the 30‐day period was significantly associated with higher baseline depression scores and higher baseline risky drinking behaviors. Binge drinking was significantly more likely to occur at someone else's house compared to other locations, though it was not associated with time of day or social group size. Furthermore, lower perceived social support (ISEL appraisal) was significantly associated with a higher likelihood of consuming hard alcohol, and drinking with two or more people significantly increased the odds of hard alcohol use compared to drinking alone.

**Conclusion:**

Utilizing EMA is a feasible method for capturing detailed data on drinking behaviors and context with American Indian women. The findings highlight the critical role of social setting, social support, and underlying depressive symptoms in driving binge drinking and beverage choice, offering valuable insights for the development of tailored interventions.

## Graphical abstract

To track real‐time alcohol use, 40 American Indian women completed a 30‐day Ecological momentary assessment (EMA). Results revealed that binge drinking during the 30 days was linked to baseline depression, risky drinking behaviors at baseline, and when alcohol was consumed at someone else's house. Additionally, lower social support and drinking with a group increased hard liquor consumption. EMA proved feasible, demonstrating that social context and social support drive drinking behaviors—offering key insights for developing tailored interventions with this population.

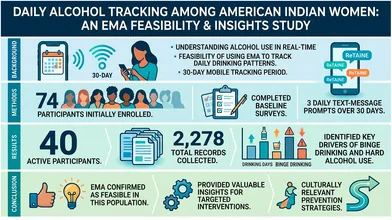

## 1 Introduction

Alcohol consumption has remained a common social practice within many communities across the globe (World Health Organization 2018). One subpopulation that has specifically seen an increase in risky drinking patterns over the past 25 years, including increased binge drinking (four or more drinks on a single occasion) and heavy drinking (eight or more drinks per week), is women (National Institute on Alcohol Abuse and Alcoholism, n.d.). In the United States, binge drinking is particularly increasing among women in their 30s and 40s (Keyes et al. 2019), with approximately 18.4% of women 18 years of age and older reporting binge drinking in the past month (Samhsa 2024). Women also face a higher probability of alcohol‐related medical problems compared to men, including cardiovascular diseases and diabetes (World Health Organization 2014), liver conditions (Eagon 2010; Rehm et al. 2010), alcohol‐induced cardiomyopathy (Fernández‐Solà et al. 1997; Fernández‐Solà and NICOLÁS‐Arfelis 2002; Urbano‐Márquez et al. 1995), specific types of cancer (Allen et al. 2009), and psychiatric comorbidities, including anxiety and depression (Benishek et al. 1992; Boykoff et al. 2010; Goldstein et al. 2012; King et al. 2003; Parks et al. 2001; Wu et al. 2010). Moreover, women may progress more quickly from regular drinking to problematic alcohol use than men, leading to a shorter time between initial use and the need for treatment—a pattern referred to as “telescoping” (Alvanzo et al. 2011; Hernandez‐Avila et al. 2004; Johnson et al. 2005; Lewis and Nixon 2014).

Indigenous communities within the US, including American Indian communities, represent another subpopulation that has received increased attention regarding alcohol consumption (Duran 1996; Wendt et al. 2019). A study examining drinking behavior among American Indian women before conception and during pregnancy found that they consumed less alcohol prior to conception when compared to White women, thereby “challeng[ing] commonly held beliefs of elevated alcohol consumption among American Indians compared with other races” (Specker et al. 2018). Research has also found that American Indian women have a higher probability of abstaining from alcohol when compared to other racial/ethnic groups, although when they drink, they are more likely to binge drink when compared to non‐American Indian women (Ye et al. 2020). Other research supports this, finding lower rates of substance abuse among many tribal communities when compared to the general US population, including higher rates of alcohol abstinence (Beals et al. 2003; Cunningham et al. 2016; Spicer et al. 2003; Whitesell et al. 2012). A national study similarly showed that while American Indians were more likely to consume alcohol at risky levels, Whites had twice the odds of current drinking, with no difference between other racial/ethnic groups and American Indians (Greenfield et al. 2021). There is also significant variability in drinking rates when comparing different American Indian/Alaska Native (AI/AN) tribes, as Whitesell et al. (2012) note, “It is clear that substance use and disorder threaten the health of AI/AN communities, yet neither are universally pervasive within these communities.”

The methods to collect alcohol drinking data among tribal communities has typically been completed through retrospective data collection, such as through the utilization of the Timeline Followback (TLFB) (Dukes et al. 2017; Ye et al. 2020), cross‐sectional surveys about past binge drinking (Hanson et al. 2017; May and Gossage 2001), and open‐ended questions (e.g., establishing the specific types of alcohol consumed, size of containers, number of individuals sharing, and timeframe of drinking episodes) (Hanson et al. 2017; Ye et al. 2020). However, these methods may not provide accurate data on predictors or clear patterns of drinking behavior because of retrospective recall issues (Kaplan and Koffarnus 2019; Shiffman 2009). Furthermore, there are few research studies that examine non‐drinking days among adult American Indian women, which may aid in understanding protective factors against risky drinking to develop targeted interventions. Hence, additional data on the contextual variables of drinking (e.g., type of alcohol consumed, setting and group size during drinking, and other psycho‐social variables such as mood during a drinking episode) is necessary to gain deeper insight into the drinking patterns and to inform interventions aimed at reducing risky drinking among American Indian communities (Treloar et al. 2015; Witkiewitz et al. 2012).

To meet these needs, ecological momentary assessment (EMA) is an effective method for capturing richer contextual detail around alcohol use, as it prompts participants multiple times per day through cell phones or other digital devices to report their behaviors in real time and within their natural environments (Shiffman 2009; Shiffman et al. 2008). EMA helps to produce detailed data on the environmental factors that shape drinking behavior in the moment it happens (Shiffman 2009). As Shiffman (2009) further emphasizes, EMA is especially effective for studying substance use because it can collect an individual's mood and surrounding context during specific drinking episodes. By reducing the bias linked to retrospective self‐reporting, EMA may offer more accurate insights into binge drinking patterns—including the types of alcohol consumed, drinking triggers, and high‐risk situations—while also capturing data from non‐drinking days, which may reveal potential protective factors (Shiffman 2009).

EMA has been widely used among general populations to better understand drinking behaviors (Serre et al. 2015; Votaw and Witkiewitz 2021). Specifically, it has been used with college‐aged students to better understand binge drinking patterns and co‐occurring behaviors (Gius and Schlauch 2021; Howe et al. 2022; Hultgren et al. 2020; Phillips et al. 2022; Stevens et al. 2020; Yang et al. 2022) and with high‐risk populations, such as women with mental health issues (Jahng et al. 2011). Further, EMA has been used to develop a better understanding of the impact of risky drinking, such as research on hangovers and alcohol‐impaired driving (Epler et al. 2014; Motschman et al. 2020; Piasecki et al. 2012; Robertson et al. 2012). However, EMA has not yet been fully utilized for better understanding drinking behaviors within American Indian communities.

Therefore, the purpose of this study was to utilize EMA with American Indian women to better understand drinking patterns and the situational context in which drinking occurs, both to determine protective factors and to inform interventions to reduce risky drinking in this population. Previous research from this team showed that EMA was feasible and acceptable for use with American Indian women (Hanson et al. 2023) but that a larger sample size was necessary to better understand the drinking style, preferred alcohol type, risky days/times of drinking, mood and affect prior to and during drinking episodes, and the social context in which drinking occurs.

## 2 Materials and Methods

This study was approved by the Human Subjects Institutional Review Board (IRB) at the University of Minnesota, the participating community's Tribal Council, and the Great Plains Indian Health Service (IHS) IRB. We previously tested the feasibility and acceptability of EMA within a tribal community (Hanson et al. 2023). In order to validate our data collection tool and the data collection system itself, we conducted formative research via think‐alouds (Fonteyn et al. 1993) with 20 American Indian women, who provided feedback both on the baseline survey and on the EMA survey. Feedback, such as the wording of some of the questions, was incorporated into the surveys. We then tested the data capture systems with 12 individuals for quality assurance purposes prior to enrolling any participants.

### 2.1 Eligibility and Recruitment

To determine eligibility for the study, individuals first completed a screening survey. Eligibility included those who self‐identified as American Indian or Alaska Native, were between 18 and 50 years old, identified as female, were not pregnant at the time of enrollment, and had consumed more than one alcoholic drink in the past month. Recruitment efforts were led by a local Project Coordinator—a member of the target community—who distributed flyers at clinics, nonprofit and community organizations, and local businesses. Word‐of‐mouth also played a key role in outreach. Interested individuals contacted the Project Coordinator directly, who then conducted eligibility screening. Those who were eligible provided written consent for participation and were provided with a copy of the signed consent form to keep. Upon participant request, the Project Coordinator was available to meet with participants in person to discuss concerns throughout the study. To assist with data collection, participants were offered a telephone calling card or a project TracFone, which they were allowed to keep after project completion.

### 2.2 Measurements and Data Collection

Baseline data were collected in person from each participant. This included the following assessments: demographic questions; a 30‐day TLFB (Dukes et al. 2017; Sobell and Sobell 1992); the Alcohol Use Disorders Identification Test (AUDIT) (Babor et al. 1992); motives for drinking via the Drinking Motives Questionnaire (DMQ) that included subscales on drinking for social, coping, enhancement, and conformity reasons (Kuntsche and Kuntsche 2009); questions on social support using the Interpersonal Support Evaluation List (ISEL) that included subscale measures of belonging, appraisal, and tangible support (Cohen et al. 1985); and questions on mental health using the Center for Epidemiologic Studies Depression Scale—Revised (CES‐D‐R‐10) (Björgvinsson et al. 2013; Radloff 1977). These baseline data were entered into the Research Electronic Data Capture (REDCap) system.

To enroll in the EMA portion of the study, each participant's cell phone number was forwarded to the principal investigator to be entered into the ReTAINE (Real Time Assessment in the Natural Environment) system, an automated internet‐based system for designing, administering, and managing EMA that has previously shown high participant adherence rates (Engel et al. 2013). RETAINE is hosted on a Microsoft Azure virtual dedicated environment and is protected by 2048 bit SSL encryption for all participant‐submitted data. The application utilizes dual authentication to servers behind a network firewall. This system sent automated signals to the participant's cell phone via SMS text. The fixed signals occurred three times daily at 11 am (morning signal); 7 pm (first evening signal); and 11 pm (second evening signal) with an individual assessment link that read: “please complete. https://retaine.org/Assessment…”. The link brought participants to the ReTAINE system to answer survey questions for data collection.

Daily data collection via the three signals began by asking the participant if they had a drink of alcohol since the last report (yes/no). If the response was “yes,” they were asked the following:

1. What they drank and drink quantity (total number of standard drinks and type of drink), with definitions of standard drinks from the National Institute on Alcohol Abuse and Alcoholism (National Institute on Alcohol Abuse and Alcoholism 2025) included;
2. Perceived drink effects (pleasurable, drink relieved unpleasant feelings);
3. Location where the drinking occurred (home, other private residence, school, work, restaurant, bar, other);
4. Social context in which the drinking occurred (alone, with one other person, in a group with more than two people); and
5. If other substances were used during the drinking episode (e.g., tobacco or other drugs).

As part of the morning signal, participants were asked if they had any unreported data from the previous night. If they did, they could add this information at that time, helping to fill in any missing data. Participants were able to skip any questions they did not want to answer; there were no questions that required a response before moving on to the rest of the questionnaire. Regardless of drinking behaviors, every participant was asked about positive and negative affect via the *Positive and Negative Affect Schedule* (PANAS‐SF) (Watson et al. 1988).

We collected data in this way with each participant for 30 days. No identifiable participant information was shared or displayed on automated text message signals, which requested completion of questionnaires. After 30 days of signals, participants completed a 30‐day TLFB to validate the previous data collection. Those who completed 35% of the prompt surveys over the 30‐day data collection period received a $40 gift card and were entered into a drawing to receive one of three $100 gift cards.

### 2.3 Statistical Analysis

Baseline data were analyzed using descriptive statistics. The scale scores for the AUDIT, DMQ, ISEL, and CES‐D were calculated as the sum of the respective items on each scale. For EMA‐specific data, the analysis was focused on participants who completed 35% of the prompt surveys and merged for each participant across the 30 days of project data collection. Categorical drinking data, including affect, type of alcohol consumed, perceived drink effects, location of drinking, social context, and use of other drugs were aggregated and averaged using basic descriptive statistics. Participants reporting four or more standard drinks on a single occasion during the study period were considered binge drinkers. The number of binge drinking occasions (e.g., greater than or equal to four drinks in a single setting) was also summed for a single participant. Chi square tests of independence were used to assess the frequency of type of drinking episode (binge yes/no) and the association with demographics. The relationships between setting (time of day, social context, location), ISSEL subscores, and the use of different types of alcohol during drinking occasions were examined using mixed‐effects logistic regression models, looking for the presence or absence of each type of alcohol while controlling for context, such as time of day, who the participant is drinking with, and where the drinking took place, as well as social support, with random effect terms for participant to account for repeated measures. Results were summarized using odds ratios with 95% confidence intervals and *p*‐values.

Analyses were conducted using SAS Version 9.4 (SAS Institute Inc., Cary, NC, USA) and R version 4.4.2 (R Foundation for Statistical Computing, Vienna, Austria).

## 3 Results

### 3.1 Baseline Data

A total of 74 participants consented to participate, had baseline data collected, and had their contact information uploaded into the ReTAINE system. The average age of participants was 35.7 years (SD = 10.86, range 18–65). Table 1 provides demographics from the baseline survey. The average from the ISEL scale was 26.80 (SD = 2.69, range 18–30), with averages from the subscales as follows: belonging subscale average was 9.56 (SD = 1.78, range 5–14); appraisal subscale average was 9.17 (SD = 2.04, range 3–12); and tangible subscale was 8.06 (SD = 1.17, range 5–12). The average from the CES‐D was 9.29 (SD = 4.86, range 0–20), with *n* = 29 participants (43.28%) scoring 10 or higher, indicating possible depression (*n* = 7 were excluded due to missing responses).

**TABLE 1:** Demographic description of participants (*N* = 74).

Variable (# missing)	*N* (%)
Marital status (0)	
Single	37 (50)
Married/partner	26 (35)
Previously married	11 (15)
Employment (1)	
Employed	47 (65)
Unemployed	22 (30)
Other/unable to work	4 (5)
Education (1)	
More than a high school education	35 (48)
High school graduate	33 (45)
Less than high school	5 (7)
Household income (0)	
Less than $5000	22 (30)
Between $5000 and $50,000	35 (47)
More than $50,000	17 (23)

Results from the AUDIT were available for *n* = 61 participants, with data missing from the rest. The mean AUDIT score was 11.91 (SD = 7.42, range 2–31). Low‐risk alcohol consumption (scores of 1–7) was found in *n* = 23 (37.70%) respondents; hazardous/harmful alcohol consumption (scores of 8–14) was found in *n* = 18 (29.51%) respondents; and possible alcohol dependence (scores of 15 or more) was found in *n* = 20 (32.79%) respondents. Baseline TLFB data across 30 days, 63.47 drinks (SD = 69.29, range 0–307) were consumed with an average of 2.13 (SD = 2.24, range 0–9.9) drinks per occasion. Averages from the DMQ subscales were as follows: social subscale average was 13.39 (SD = 4.37, range 5–25); coping subscale average was 10.25 (SD = 3.86, range 4–23); enhancement subscale average was 10.62 (SD = 3.91, range 4–23); and conformity subscale average was 11.91 (SD = 4.06, range 5–22).

### 3.2 EMA Data

Of the 74 participants enrolled, *n* = 40 completed at least 35% of EMA prompts. Table 2 includes means and SD for baseline demographic measures, comparing the completers of the EMA prompts with those who did not complete the EMA prompts. When comparing participants who completed the EMA prompts versus those that did not, EMA completers were older (*p* = 0.01). EMA completers also trended toward greater belongingness on the social support ISSEL scale (*p* = 0.08). Differences in hazardous drinking (via the AUDIT), consumption of standard drinks (via TLFB), and drinks per occasion were non‐significant between EMA completers and non‐completers. Depression symptoms were also non‐significant between EMA completers and non‐completers.

**TABLE 2:** Baseline demographic characteristics (*N* = 74).

Variable	EMA completer (*N* = 40)		Baseline assessment only (*N* = 34)	
	Mean (SD)	Range	Mean (SD)	Range
Age^a^	38.48 (10.19)	21–65	32.31 (10.8)	18–65
AUDIT	11.94 (7.43)	4–31	11.86 (7.57)	2–24
Total drinks	69.65 (72.11)	0–268	56.00 (66.03)	0–307
Drinks/occasion	2.36 (2.32)	0–8.64	1.86 (2.31)	0–9.90
Depression symptoms	8.94 (4.85)	0–19	9.75 (4.93)	3–20

^a^ Significant effect of age, *p* = 0.01. *AUDIT*: Alcohol Use Disorders Identification Test (AUDIT) (Babor et al. 1992); *Total Drinks*; *Drinks/Occasion*: 30‐day Timeline Followback (TLFB) (Dukes et al. 2017; Sobell and Sobell 1992); *Depression Symptoms*: Center for Epidemiologic Studies Depression Scale—Revised (CES‐D‐R‐10) (Radloff 1977).

For EMA completers, 2278 records were completed across 359 drinking days and 1919 non‐drinking days. Drinks per occasion across the 30‐day period averaged 9.12 (SD = 10.24, range 1–47). Forty‐nine percent of drinking occasions included beer, followed by hard liquor (32%), malt liquor (13%), wine (5%), and other (1%). Binge drinking throughout the 30‐day period was significantly associated with baseline depression scores (*p* < 0.05); risky drinking as assessed via the baseline AUDIT (*p* < 0.01); and the TLFB both at baseline (*p* < 0.05) and at the 30‐day assessment (*p* < 0.0001). Drinking motives and social support, collected at baseline, were not associated with binge drinking across the study period. When looking at other substance use during drinking episodes, of the 345 records, participants reported using other substances on 239 occasions (69%) and not using any other substances on 106 occasions (31%). When participants reported the specific other substances consumed (25 reports), chewing tobacco was reported most often (15 reports; 60%), followed by cigarettes (5 reports; 20%), and e‐cigarettes (3 reports; 12%).

When assessing the association between binge drinking and context (e.g., time, location, and social context), results showed that binge drinking was associated with location, *Χ* ^2^ = 16.83, *p* = 0.002, meaning that it was more likely to occur at someone else's house (23% of episodes for binge drinking versus 7% of episodes for non‐binge drinking). In comparison, non‐binge drinking was more likely to occur in a restaurant or other location. Binge drinking was not associated with social context (e.g., drinking alone, with one other person, or in a group) or with other substance use, nor was it associated with the time binge drinking occurred (11 am [34.17% reported binge drinking]; 7 pm [32.50%]); and 11 pm [33.33%] compared to non‐binge drinking reporting (32.38%, 39.05%, and 28.57%, respectively).

Next, we looked at occasions when drinking was reported (359 occasions among 37 participants) and examined the influence of factors such as setting and social support on the use of specific types of alcohol. The three most frequently observed types (beer, 195 occasions; hard alcohol, 127 occasions; malt beverage, 52 occasions) were modeled as outcomes in multivariable logistic regression models; wine (19 occasions) and other (13 occasions) were considered too infrequent for multivariable modeling. Results are reported in Table 3. After adjusting for other factors, two scores were associated with being less likely to use hard alcohol during a drinking occasion: higher ISSEL appraisal scores (OR per 1‐point change = 0.39 [95% CI 0.16–0.94], *p* = 0.04) and higher ISSEL tangible scores (OR = 0.35 [0.09–1.34], *p* = 0.12). Participants with higher ISSEL tangible scores also tended to be more likely to use beer during a drinking occasion (OR = 5.08 [0.76–33.8], *p* = 0.09). Drinking with one other person, compared to drinking alone, tended to be associated with increased use of hard alcohol (OR = 2.55 [0.77–8.49], *p* = 0.13) and beer (OR = 3.98 [0.73–21.8], *p* = 0.11); drinking with two or more other persons was also associated with increased use of hard alcohol (OR = 9.13 [2.39–34.9], *p* = 0.001). Locations other than “my house” were less likely to have hard alcohol use including “restaurant” (OR = 0.10 [0.01–1.18], *p* = 0.07) or “other” (0.17 [0.03–0.83], *p* = 0.03).

**TABLE 3:** Multivariable logistic regression examining associations between social support measures and setting and use of different types of alcohol during drinking occasions.

	Outcome: Hard alcohol		Outcome: Beer		Outcome: Malt	
	OR (95% CI)	*p*	OR (95% CI)	*p*	OR (95% CI)	*p*
Time						
7 pm vs. 11 am	0.98 (0.39, 2.46)	0.96	1.00 (0.33, 3.03)	0.99	0.46 (0.12, 1.78)	0.26
11 pm vs. 11 am	0.99 (0.37, 2.64)	0.98	1.97 (0.56, 6.90)	0.28	0.57 (0.13, 2.45)	0.45
ISSEL subscore						
Appraisal (per 1 pt)	0.39 (0.16, 0.94)	0.04	0.98 (0.31, 3.12)	0.98	0.81 (0.33, 2.03)	0.66
Belonging (per 1 pt)	0.89 (0.44, 1.80)	0.74	0.62 (0.24, 1.58)	0.32	1.88 (0.88, 4.02)	0.10
Tangible (per 1 pt)	0.35 (0.09, 1.34)	0.12	5.08 (0.76, 33.8)	0.09	0.27 (0.05, 1.40)	0.12
Who drink with						
One other vs. Alone	2.55 (0.77, 8.49)	0.13	3.98 (0.73, 21.8)	0.11	0.81 (0.16, 4.15)	0.80
Two or more vs. Alone	9.13 (2.39, 34.9)	0.001	1.98 (0.37, 10.6)	0.43	2.70 (0.52, 14.2)	0.24
Location						
Bar vs. my house	0.68 (0.17, 2.69)	0.58	3.03 (0.47, 19.4)	0.24	0.28 (0.04, 1.91)	0.19
Other vs. my house	0.17 (0.03, 0.83)	0.03	3.65 (0.50, 26.6)	0.20	1.18 (0.13, 10.91)	0.88
Restaurant vs. my house	0.10 (0.01, 1.18)	0.07	0.67 (0.02, 23.0)	0.83	0.78 (0.03, 18.4)	0.88
Someone's house vs. my house	0.38 (0.10, 1.48)	0.16	4.06 (0.56, 29.7)	0.17	0.17 (0.02, 1.25)	0.08

## 4 Discussion

Besides our previous EMA feasibility and acceptability pilot study with an American Indian community (Hanson et al. 2023), this paper describes the results of the first known implementation of alcohol‐specific EMA with an American Indian community. EMA has been used broadly with various populations and for many health behaviors, including alcohol use (Epler et al. 2014; Gius and Schlauch 2021; Howe et al. 2022; Serre et al. 2015; Shiffman 2009; Treloar et al. 2015; Votaw and Witkiewitz 2021). The motivating factors for this project were the need to learn more about both drinking and non‐drinking days within this community and to understand the context in which drinking happens with American Indian women. For example, interventions can be built based on specific drinking contexts, such as addressing those who tend to binge drink in large groups and/or responding to mood and affect prior to and during drinking episodes. Based on the significant association between the 30‐day EMA data collection and the baseline AUDIT and TLFB results, EMA was a valid way of collecting alcohol data with American Indian women.

Many of the results of this study confirm what is already known in the published literature, adding to the breadth of literature on alcohol use with American Indian communities. In our study, participants had more non‐drinking days than drinking days, similar to other studies that showed American Indian women were more likely to abstain from alcohol when compared to White women (Ye et al. 2020). Also, like other research findings (Beals et al. 2003; Greenfield et al. 2021; Hanson et al. 2013; Vaeth et al. 2017; Whitesell et al. 2012; Ye et al. 2020), our study showed that when American Indian women drank, they tended to drink at bingeing levels, with drinks per occasion averaging 9.4, and with binge drinking significantly related to baseline depression scores. While this study's baseline AUDIT results showed that approximately 38% of the sample could be classified as low‐risk drinkers, this is likely due to complete alcohol abstinence. More specifically, there were no reports of low‐risk alcohol consumption in those who completed the EMA part of this study—all reported either hazardous/harmful alcohol consumption (31.7%) or alcohol dependence (68.3%).

One of the main goals of conducting this study was to better understand the context in which drinking is occurring with this population. As was noted earlier, much of the data on drinking with American Indian communities comes from retrospective data collection, with little focus on the context in which this drinking occurs. Binge drinking was significantly associated with location and was more likely to occur in private spaces—at someone else's home—rather than in public spaces, such as a restaurant. While binge drinking was not associated with social context, as in who the person was drinking with or what time the drinking occurred, drinking with others also increased types of alcohol consumed in that drinking with at least one other person compared to drinking alone significantly increased use of hard alcohol and beer. These data help capture predictors or clear patterns of drinking, which can be important in developing interventions.

There are some limitations to this study. First, we also do not know why approximately half of all those that consented to participate did not subsequently provide adequate data to the EMA prompts. It is possible this relates to the large number of prompts during the study (e.g., three times per day for 30 days). The EMA completers were significantly more likely to be of older age than the non‐completers, but there were no other significant differences in those that completed versus did not complete EMA. Another limitation is that this was also a relatively small sample size from one tribal community, thus the results cannot necessarily be generalized to all American Indian women. There were also high rates of depression reported at baseline in our sample, and the association between depression and alcohol dependence has been established, meaning our results are potentially biased (Kuria et al. 2012). Finally, there was no additional data collection with participants if they clicked “no” to drinking since their last response, so theoretically participants could just click “no” every time to avoid additional data collection. Asking a better initial question would aid in avoiding this issue. We also believe we are missing data on other substance use with drinking based on the limited amount of data on other substance use provided by our sample.

Regardless, EMA has been shown to be an important and valid data collection tool to reliably assess drinking in American Indian communities with significant potential to address key concerns about the context in which drinking occurs with this population. The findings highlight the critical role of drinking location, social support, and underlying depressive symptoms in driving binge drinking and beverage choice, offering valuable insights for the development of tailored interventions. Much of what was found in this study confirms other studies, although additional efforts need to be made to reach younger populations and those without depression. Data such as these can be utilized to develop public health interventions that incorporate approaches such as ecological momentary interventions (EMI), personalized feedback, behavioral health messaging, or encouragement of behavior at predefined moments in natural daily life (Beres et al. 2021; Wright et al. 2018, 2020). Based on data from this project, utilizing EMA and EMI focused interventions with tribal communities is a critical area to pursue.

## Funding

This work was supported by the National Institutes of Health, 1R15AA030673, UM1TR0044005.

## Conflicts of Interest

The authors declare no conflicts of interest.

## Data Availability

The datasets generated and analyzed during the current study are not publicly available due to tribal sovereignty regulations. Data can be made available by contacting the corresponding author and by formally applying to the relevant tribal councils and tribal review boards for access.
